# What are they returning to? Psychosocial work environment as a predictor of returning to work among employees in treatment for common mental disorders: A prospective observational pre–post study

**DOI:** 10.1371/journal.pone.0215354

**Published:** 2019-04-24

**Authors:** Bjørn Lau, Olga Shiryaeva, Torleif Ruud, Mattias Victor

**Affiliations:** 1 Lovisenberg Hospital, Nydalen, Oslo, Norway; 2 University of Oslo, Department of Psychology, Oslo, Norway; 3 Akershus University Hospital, Division Mental Health Services, Lørenskog, Norway; 4 University of Oslo, Institute of Clinical Medicine, Oslo, Norway; Erasmus Medical Center, NETHERLANDS

## Abstract

**Introduction:**

Long-term sick leave and disabilities due to common mental disorders are challenging for society, employers, and individuals. Hence, we wanted to investigate whether psychosocial work environments experienced by employees undergoing treatment for such disorders was associated with return to work.

**Methods:**

At the start of treatment, 164 patients responded to questionnaires concerning their psychosocial work environment (the Job Demand–Control–Support model and the Effort–Reward Imbalance model), symptoms (The Clinical Outcomes in Routine Evaluation Outcome Measure) and ability to work (Work Ability Index). In addition, the respondents reported whether they were working or on sick leave at the start and end of their courses of treatment. Their therapists provided information about diagnoses.

**Results:**

Return to work was associated with control of decisions, support from colleagues, esteem, and job promotion opportunities as measured at the start of treatment. In multivariate analyses, control over decisions and job promotion opportunities continued to predict return to work when adjusted for symptoms, current work ability, and expected future work ability.

**Discussion:**

The working conditions that predicted return to work are considered to facilitate work performance and to be sources of motivation, job satisfaction, and job commitment. Consequently, it is important to examine whether this patient group has a favorable working environment and consider changes in the workplace if the environment is not favorable.

## Introduction

Common mental disorders (CMDs), such as anxiety and depression, are found in a sizable proportion of people on long-term sickness and disability benefits in many Western countries [1–3]. However, participation in work was shown to have positive effects on mental health and well-being, especially under favorable workplace conditions and with good management [4]. Therefore, it is important to know why some employees with CMDs lose a potentially positive force in their lives by going on long-term sick leave or disability benefits, while others remain at work or return to work (RTW) after a period of sickness absence.

Several studies have investigated conditions related to returning to work among employees with mental disorders. In a systematic review, Cornelius et al. [5] found that several personal, health-related, and external job-related factors had impacts on long-term disability because of poor mental health. Among the health-related factors, anxiety and depression disorders were associated with longer durations of disability. In another systematic literature review with a particular focus on depressed employees, strong support was found for an association between long depressive episodes and disability. In addition, severe depressive symptoms were associated with work limitations, while clinical improvement was related to increased labor productivity [6].

Based on the implicit idea that clinical improvement leads to better work function and thus RTW, the effect of different treatments on RTW among employees on sick leave with CMDs was investigated. A review found no support for the view that medication alone or improved primary care reduced disability in depressed employees [7]. In addition, there was no evidence for or against the effectiveness of psychological interventions to reduce disability in depressed workers. In another systematic review, [8] found that cognitive behavioral therapy did not reduce either time to partial RTW or time to full RTW, compared with no treatment among employees with adjustment disorders. Problem-solving therapy significantly improved partial RTW in a one-year follow-up period compared with care without such guidelines, but did not lead to full RTW in that period. However, a recent published systematic review and meta-analysis based on 45 studies of people on sick leave due to CMDs found that psychological interventions were somewhat more effective than care as usual on reduced sick leave, but that this effect was small [9]. Others have found promising results in the form of work-focused cognitive behavioral therapy that integrates work aspects into the treatment at an early stage [10]. This indicates that workplace conditions have importance for RTW among employees in treatment for such conditions.

In this context, research studying the relevance of working conditions for employees with CMDs ending up with disability benefits is of interest. In a systematic review, [11] found that lack of support at the workplace, alone or in combination with high work demands, increased the risk of going on disability benefits. They also found studies that showed that an imbalance between the effort made at work and the reward received over time increased the risk of becoming disabled. Also, a review by Cornelius et al. [5] showed that management support in the form of frequent communication between employees and managers was associated with reduced duration of disability. However, this was not a comprehensive finding, and in one study, such a connection was found only among employees with minor depression [7]. In another study, management support was even found to be somewhat negatively associated with RTW among employees with mental health problems [12]. Consequently, although support in the workplace usually improves the likelihood of returning to work [13,14], it is suggested that employees with more severe mental health problems such as depression may benefit less from communication with their leaders [12] [7].

Other studies have investigated the effect of working conditions on RTW among employees on sick leave due to CMD. Various studies have identified factors such as shorter work weeks [15], employment in a larger organization [16], and variation in work [17]. Additionally, research has shown that supervisor support [18], good communication with leadership [19], and experience of interactional justice with their supervisor [20], in addition to job recognition [18] and various forms of colleague support [15,18] are positively related to RTW. Similarly, both high decision latitude [15] and low decision authority [18] were found to be associated with RTW. One study also found a weak negative relationship between high job demands and RTW [18].

Based on these findings, we wished to examine the associations between work-related conditions and RTW among patients who had undergone treatment for CMDs. We focused on work-related dimensions taken from models that have shown to be of importance for the development of poor mental health in previous studies [21], such as the Job Demand–Control (JDC) model [22] and the Effort–Reward Imbalance (ERI) model [23]. In these models, high job demands are considered a potential risk for the development of poor health, especially when combined with low control and/or support (JDC model) or in combination with the lack of various forms of rewards, such as esteem, career opportunities, or job security (ERI model). In a later developed generic model, the Job Demand–Resource model, the starting point is that all aspects of a job can be classified as either job demands or job resources [24]. Job demands are physical, mental, social, or organizational conditions requiring sustained physical or mental efforts; therefore, they are associated with physiological or psychological costs [25]. Job resources are physical, mental, social, or organizational aspects of work that are functional in the performance of the work demands, but also stimulate personal growth, learning, and development [24,26].

In the present study, we decided to analyze the same sample with patients treated for CMDs that we previously examined with prospective observational data to determine whether treatment-related factors and other individual factors affect RTW [27]. We assumed that a work environment characterized by manageable tasks and good resources would have a positive impact on RTW. Consequently, we produced a series of hypotheses that different types of job demand in the previously mentioned models (i.e., quantitative, decision, and learning demands and efforts) would be negatively associated with RTW. Similarly, we produced a series of hypotheses that various assumed job-related resources (control of decisions, control of work pacing, support from superior, support from colleagues, esteem, job promotion, and job security) would be positively associated with RTW. In these analyses, we also controlled for other work-related factors as previously found in the same material to affect RTW, such as self-reported current and expected future work ability, as well as symptoms at the baseline [27].

## Material and methods

The study was approved by the Regional Committee for Medical and Health Research Ethics, South East Norway (case number 2010/494), and the Lovisenberg Hospital management (case number 03–2010 LDS). The study was conducted according to the principles of the Helsinki Declaration and informed consent was obtained from all patients included in the study.

### Design and treatment setting

The work participation changes were examined with a prospective observational pre–post study design among patients at an RTW clinic in a community mental health center. Successful RTW was identified by changes in work participation. Work conditions at baseline, in addition to symptoms, present work ability, and future work ability expectancy were used to predict RTW.

In the RTW clinic, patients are referred by general practitioners (GPs) to clinical psychologists for treatment for their mental health problems. Patients must be working and have the right to sickness benefits. They can be on sick leave, or in their GP’s opinion be in danger of becoming so. Therefore, the program’s aims are to return patients on sick leave to work and to prevent sick leave for those still working.

No modifications in treatment practice or extraordinary inclusion or exclusion criteria were adopted for this study. All new patients were eligible to participate. Participants were recruited during 10 consecutive months (August 16, 2010, to June 15, 2011) from patients who attended their first session at either the RTW clinic or the regular outpatient clinic. The offered treatment was individual, time-limited psychotherapy and/or psychoeducational courses for various problems, such as stress, depression, panic disorders, social phobia, and insomnia. Of the 164 patients answering the questionnaire at the beginning and end of treatment in our study, 109 (67%) received individual psychotherapy, 21 (13%) received group interventions, and 34 (21%) received a combination of individual psychotherapy and group interventions. The average duration of treatment was 36 weeks. The average number of sessions were 20 (range 1–96) for the entire sample, and 18, 12, and 33 for patients who received individual psychotherapy, group interventions, and combined treatment, respectively. There was no significant difference in the number of consultations between those who successfully returned to work (19.4 consultations) compared with those who did not (22.6 consultations). The average time on a waiting list was 51 days (range 2–172 days).

### Participants

The participants were recruited from 561 new patients over a 10-month period. In the first data collection, 267 patients responded to the questionnaire, while 165 were not asked to participate, 119 refused to participate, and 10 patients were excluded by their therapist. Of the 267 included patients, 103 failed to send back the questionnaire when their treatment had finished. Therefore, the final sample included 164 patients with complete questionnaires before and after treatment. No selection or dropout bias was found when medical records of the patients included in the first data collection (*n* = 267) were compared with those of the other eligible patients (*n* = 294) in terms of age, sex, therapeutic diagnoses, or Global Assessment of Functioning (GAF) scores, nor was bias detected in comparisons of the 164 patients who returned the questionnaire at both data collection points with the 103 patients who provided only initial responses. For more details, see [27].

Background variables characterizing the sample at baseline are shown in Table 1. The mean age was 38.2 years (SD = 10.4 years) and 69% reported graduating from college or university. Patients were diagnosed according to the International Classification of Diseases 10 (ICD-10). The diagnoses were grouped into five categories: 1) depression (depressive episodes [F32] and recurring depressive episodes [F33]); 2) anxiety (phobic anxiety disorders [F40], other anxiety disorders [F41], and obsessive–compulsive disorder [F42]); 3) adjustment disorders [F43]; 4) other psychiatric diagnosis (e.g., substance abuse and eating disorders); and 5) Z-diagnoses (i.e., contacts with health services for reasons not resulting in psychiatric diagnoses, such as examinations). However, for a group of patients (n = 18) psychiatric diagnoses was not registered in their journal. This group were mainly patients who only met for psychoeducational courses and were not individually diagnosed by their group leader. The diagnosis type was not used as a predictor or covariate of RTW in the analyzes because a previous study with the same sample showed that they could not predict RTW [27].

**Table 1:** Patient characteristics at baseline according to RTW.

		All		Successful RTW		Failed RTW	
		N	%	N	%	N	%
Total		164		117	71%	47	29%
Age	18–29	33	20%	23	20%	10	21%
	30–39	69	42%	51	44%	18	38%
	40–49	35	21%	26	22%	9	19%
	50–	27	17%	17	15%	10	21%
Sex	Women	116	71%	80	68%	36	77%
Marital status	Living with partner	84	52%	61	53%	23	49%
Education	Comprehensive school (1–9 years)	11	7%	6	5%	5	11%
	Secondary/vocational school (10–12 years)	40	25%	29	25%	11	23%
	College degree (13–16 years)	78	48%	53	46%	25	53%
	Higher university degree (>16 years)	34	21%	28	24%	6	13%
Main diagnosis (ICD-10)	Depression (F32–F33)	65	45%	44	43%	21	48%
	Anxiety (F40–F42)	31	21%	23	23%	8	18%
	Adjustment disorder (F43)	34	23%	24	24%	10	23%
	Other psychiatric diagnoses	12	8%	7	7%	5	11%
	Z-diagnoses	4	3%	4	4%	0	

### Measurements

#### Outcome variables: Work participation and RTW

A work participation index with three reciprocally exclusive categories was created based on data from patients, therapists (questionnaires), and medical files. The categories were: working fully, part-time work (part-time work in addition to sick leave, a social welfare benefit, or partial unemployment), and not working (mostly on full sick leave). By combining these three categories from the pre- and posttreatment periods, a new dichotomous variable describing RTW was created. This variable consisted of two categories: A) successful RTW based on 1) full RTW and 2) partial RTW; 3) still in full-time work; and 4) still in part-time work. The second category was labeled failed RTW and consisted of those who were 5) still not working or 6) working less.

#### Mental health

Symptoms. The Clinical Outcomes in Routine Evaluation Outcome Measure (CORE-OM) was used to measure patient-reported symptoms [28]. This measure contains 34 questions on four domains related to the previous week. All items are scored from never (= 0) to almost always (= 4). The total mean scores are usually multiplied by 10 to calculate a total score ranging from 0 to 40 [29]. Forms with fewer than 90% of the items completed were excluded from the analyses. Similarly to [30], we found a Cronbach’s α of 0.92 using baseline data. The total score was used in the logistic regression analyses.

Work ability. We used two of the original seven questions from the Work Ability Index (WAI) [31]. We did not use all seven questions because of space considerations in the questionnaire and because the first question correlates highly with the total score on the WAI [32]. The first question was: “Assume that your work ability at best has a value of 10 points. How many points would you give to your current working ability?” This question was scored from 0 (= worse) to 10 (= best). The second question was: “Considering your health, do you think you will be able to perform your current job in two years?” This question was assigned four response options: 1) Yes, definitely; 2) Yes, to some degree; 3) No, not really; and 4) No, absolutely not. A dichotomous version of this question was created for the analyses by merging the first two and last two response options.

#### Psychosocial work environment

QPSNordic. Key components from the JDC model [22], which was later developed into the Job Demand–Control–Support (JDCS) model [33], were measured using the Nordic Questionnaire for Psychological and Social Factors at Work (QPSNordic) [34]. The QPSNordic is a validated self-report measure developed to cover a variety of essential psychological and social factors at work that are important for health, motivation, and job satisfaction. The questionnaire consists of questions on the individual, social, organizational, and task levels. In this study, items from seven scales measuring job demands, job control, and work support were included. Job demands were measured on three scales: i.e., quantitative demands (four questions such as “Is your work load irregular so that the work piles up?” or “Is it necessary to work at a rapid pace?”), decision demands (three questions such as “Does your work require quick decisions?” or “Does your work require maximum attention?”), and learning demands (three questions such as “Do you perform work tasks for which you need more training?” or “Does your job require that you acquire new knowledge and new skills?”). Two scales measured control at work: i.e., control of decisions (five questions such as “If there are alternative methods for doing your work, can you choose which method to use?”, “Can you influence the amount of work assigned to you?” or “Can you influence decisions that are important for your work?”) and control of work pacing (four questions such as “Can you set your own work pace?” or “Can you decide yourself when you are going to take a break?”). Two scales measured job support: i.e., support from superior (three questions such as “If needed, can you get support and help with your work from your immediate superior?” “If needed, is your immediate superior willing to listen to your work-related problems?”) and support from colleagues (the two questions were “If needed, can you get support and help with your work from your coworkers?” and “If needed, are your coworkers willing to listen to your work-related problems?”). A five-point response scale ranging from 1 (very seldom or never) to 5 (very often or always) was used for all questions. The mean values were computed for the items in each subscale. Cronbach’s α values for the scales were: quantitative demands, 0.77; decision demands, 0.60; learning demands, 0.58; control of decisions, 0.75; control of work pacing, 0.83; support from superior, 0.90; support from colleagues, 0.83.

The Effort–Reward Imbalance questionnaire. As an alternative theoretical model, the ERI model [23] emphasizes rewards instead of the control and support structure at work. The ERI model assumes that efforts at work are partly spent as an exchange in which the workplace and society contribute to occupational rewards through three transmitter systems: i.e., esteem (e.g., respect and support), career opportunities (e.g., promotion prospects), security (e.g., job security and status consistency). In this study, we measured the effort and these three rewards with a validated Norwegian version of the standardized ERI questionnaire traditionally used to measure these parameters [35]. Five questions were included in the effort scale (e.g., “I have constant time pressure due to a heavy work load” and “I have many interruptions and disturbances while performing my job”). Three subscales measured reward: 1) esteem (five items, such as: “Considering all my efforts and achievements, I receive the respect and prestige I deserve at work”; “I receive the respect I deserve from my superiors”; and “I receive the respect I deserve from my colleagues”); 2) job promotion (four items, such as: “My current occupational position adequately reflects my education and training” or “Considering all my efforts and achievements, my job promotion prospects are adequate”); and 3) job security (two items, i.e.: “I have experienced or I expect to experience an undesirable change in my work situation” and “My employment security is poor”). The responses to the questions measuring effort were answered on a five-point Likert scale ranging from 1 (does not apply), 2 (does apply, but not strained), to 5 (does apply and very strained). The 11 elements measuring the three reward scales were set similarly, but the coding was reversed; i.e., low scores indicate stress because of low reward. The mean values were computed for the items in each subscale. Cronbach’s α values for the scales were: effort, 0.79; esteem: 0.81; job promotion, 0.73; and job security, 0.40.

### Statistics

Descriptive statistics (means and frequencies) were used to summarize sample characteristics and questionnaire responses. The general linear model (GLM) univariate analysis of variance (ANOVA) was used to test for mean differences in work environment variables between the successful and failed RTW groups. Effect sizes (Cohen’s *d*) were calculated, and were defined as small (*d* = 0.2), medium (*d* = 0.5), or large (*d* = 0.8) (Cohen, 1988). Logistic regression analysis was used to identify factors associated with RTW. First, univariate logistic regression analyses were performed for all work environment variables, present work ability, future work ability expectancy, and symptoms, with RTW as the dependent variable. In multivariate logistic regression analyses, the significant work environment variables identified by the univariate logistic regressions were entered in a series of multivariate logistic regression analyses, controlling for symptoms (CORE total), present work ability, and future work ability expectancy. Multicollinearity between the remaining independent variables was tested by checking the variance influence factor (VIF) statistics. Multicollinearity was assumed when the VIF scores were greater than 4, which none of the variables reached. Data were analyzed using SPSS for Windows (version 24; IBM Corp., Armonk, NY, USA).

## Results

As shown in Table 1, 117 (71%) of the sample successfully returned to work. More women received treatment and about half of the sample were married or cohabiting. The average age was 38.2 years (standard deviation [SD] = 10.4 years). In terms of education, 69% graduated from college or university. Most patients were diagnosed with CMDs (anxiety, depression, or adjustment disorders). In a series of logistic regression analyses (not shown here), no differences were found between the variables listed in Table 1, i.e., age, sex, marital status, education, or diagnosis, when these were entered as independent variables to predict RTW (successful versus failed RTW).

As shown in Table 2, patients who achieved successful RTW had significantly higher mean baseline scores on the working environment scales (i.e., control of decisions, support from colleagues, esteem, and job promotion) than the group with failed RTW. These results were revealed in a series of GLM univariate ANOVA where RTW was entered as the independent variable and the psychosocial work environment variables were entered as dependent variables in a series of separate analyses. According to Cohen’s *d*, these differences were moderate (range 0.4–0.5).

**Table 2:** Psychosocial work environment at baseline, according to RTW.

	Total		Successful RTW		Failed RTW			
	Mean	SD	Mean	SD	Mean	SD	F	*p* value
Quantitative demands	3.3	0.9	3.3	0.8	3.2	1.0	0.38	0.54
Decision demands	3.6	0.7	3.6	0.7	3.6	0.7	0.27	0.60
Learning demands	2.6	0.7	2.6	0.7	2.5	0.7	0.81	0.37
Control of decisions	2.7	0.8	2.8	0.8	2.5	0.8	6.78*	0.01
Control of work pacing	2.9	1.1	3.0	1.1	2.7	1.2	2.49	0.12
Support from superior	3.4	1.1	3.3	1.1	3.4	1.2	0.02	0.90
Support from colleagues	3.6	1.0	3.7	0.9	3.3	1.1	5.39*	0.02
Effort	2.8	1.0	2.8	0.9	2.9	1.0	0.23	0.63
Esteem	4.0	1.0	4.1	1.0	3.7	1.1	5.15*	0.03
Job promotion	3.8	1.0	3.9	0.9	3.4	1.0	6.32*	0.01
Job security	3.6	1.2	3.6	1.1	3.4	1.3	0.93	0.34

* Sig. *p* < 0.05.

Similar findings to those reported in Table 2 were found in the series of univariate logistic regression analyses shown in Table 3. In these analyses, control of decisions, support from colleagues, esteem and job promotion were all significant predictors of RTW. In addition, present work ability, future work ability expectancy and CORE total were significant predictors of RTW. Most of the presented odds ratio values were rather small, except future work ability. For this variable, the confidence interval was quite large (2–10.57) compared with the other variables, which was mainly due to many respondents in the category with successful RTW and high expectancy of RTW in 2 years (63%).

**Table 3:** Psychosocial work factors, work ability and symptoms at baseline predicting RTW in logistic regression analyses.

	OR	95% CI
Quantitative demands	1.14	0.76–1.71
Decision demands	1.14	0.69–1.89
Learning demands	1.28	0.75–2.19
Control of decisions	1.85*	1.14–2.99
Control of work pacing	1.30	0.94–1.79
Support from superior	0.98	0.70–1.35
Support from colleagues	1.53*	1.06–2.21
Effort	0.91	0.63–1.32
Esteem	1.48*	1.05–2.09
Job promotion	1.58*	1.09–2.30
Job security	1.16	0.86–1.57
Present work ability	1.24*	1.08–1.42
Future work ability expectancy	4.60*	2.00–10.57
CORE total	0.92*	0.86–0.99

* Sig. *p* < 0.05.

In a series of separate logistic regression analyses, control of decisions, support from colleagues, esteem, and job promotion were entered as predictors of RTW. In these analyses, present and future expected work ability and symptoms were controlled for. The results shown in Table 4 demonstrate that the impact of several of the working environment variables on RTW was reduced to insignificance. The exception was control of decisions, which remained significant when we controlled for present work ability and symptoms. The other exception was job promotion, which still was a significant predictor of RTW when we controlled for future work ability expectancy and symptoms.

**Table 4:** Psychosocial work factors at baseline predicting RTW in a series of logistic regression analyses: controlled for present work ability, expectancy of future work ability, and CORE total.

	Controlled for present work ability			Controlled for future work ability expectancy			Controlled for CORE total		
	OR	95% CI	NR^2^	OR	95% CI	NR^2^	OR	95% CI	NR^2^
Control of decisions	1.24*	1.07–1.44	.15	1.55	0.92–2.63	.18	1.69*	1.04–2.74	.11
Support from colleagues	1.49	0.96–2.00	.12	1.42	0.96–2.11	.19	1.43	0.98–2.08	.10
Esteem	1.34	0.93–1.93	.11	1.41	0.97–2.05	.19	1.34	0.94–1.92	.09
Job promotion	1.44	0.98–2.12	.12	1.61*	1.08–2.41	.19	1.56*	1.06–2.28	.12

NR^2^ = Nagelkerke R^2^

* Sig. *p* < 0.05.

## Discussion

In this study, we hypothesized that work-related demand dimensions (quantitative demands, decision demands, learning demands, and effort) would be negatively associated with RTW, while alleged job-related resources like autonomy (control of decisions and control of work pacing), support (support from superior and support from colleagues), esteem, job promotion, and job security would be positively associated with RTW. However, the results showed that successful RTW was only predicted by perceived control of decisions, support from colleagues, esteem, and career opportunities measured at the start of treatment. These results were supported by analyses comparing average values between those who successfully returned to work with those who did not, and by logistic regression analyses where working environment variables predicted a successful RTW.

The working dimensions in question were taken from the JDCS, ERI, and Job Demand–Resource models. Considering these models, the present results indicate that what is often considered work-related resources were associated with RTW while job demands were not. In line with our findings, the research literature finds that working environment conditions such as support from colleagues [15,18], autonomy in work [15], and job recognition [18] were associated with RTW for employees on sick-leave with CMD. However, we did not find a negative correlation between job demands and RTW as found in a previous study [18]. This may reflect that job-related demands play an important role in developing CMDs and other health-related problems, as shown in the literature [21], while job resources are of greater importance for obtaining a successful RTW. Such an interpretation would be in line with the job demand theory where job demands are seen as conditions requiring sustained efforts and are therefore associated with physiological or psychological costs [25] that have the potential to create health-deteriorating processes, where the high work demands drain employees of their mental and physical energy. This can lead to chronic weakened energy levels and health problems over time [25,36,37]. Job resources are another aspect of functional work in the performance of work demands. Resources can reduce job demands and the associated physiological and psychological costs, but can also create positive states such as job satisfaction, commitment, and motivation to work.

The dimension of control over decisions is acknowledged to moderate stress and illness in demanding work situations [38]. Control over decisions is achieved by providing the most appropriate options to resolve challenges in workplace situations. Thus, employees with a CMD who have more control over their decisions may have greater latitude to make changes and adjustments in their workplace situation to achieve a successful RTW. Another explanation could be that these employees are more strongly motivated to RTW than patients with lower levels of autonomy because their decision-making autonomy also is known to create job satisfaction, job commitment, and motivation at work [38,39]. Similarly, a supportive climate among coworkers and executives may facilitate RTW with residual symptoms and impairment because staff can receive instrumental help and support in performing specific tasks and because someone will listen and act in an emotionally supportive manner when problems are experienced. That way, support can make it easier to perform tasks, but can also make job attendance a positive experience because of the good team spirit. We believe that both explanations are valid. However, we need more research to investigate these conditions.

Esteem and career opportunities are reward parameters in the ERI model. The positive impact of these factors on RTW in our study may be the result of facilitating execution of job demands. For example, the esteem of colleagues and managers can be interpreted as appreciation of job performance. Such feedback may counteract negative ruminations and concerns connected to anxiety and depression, which makes job performance easier. However, esteem may also create positive emotions, motivation, and commitment to work. Thus, the workplace becomes a positive part of the patient’s life. Similarly, employees experiencing an elevated degree of career satisfaction, reflecting opportunities to use their skills, education, and experience in the workplace, are more likely to experience motivation and commitment to their work. Thus, a working environment with esteem and career prospects may be appreciated as an important aspect of these patients’ lives that they wish to return to.

We wanted to examine whether these significant work-related predictors could still predict RTW adjusted for other conditions that were previously found to be important for RTW in this sample [27]. However, this must not be interpreted that only variables that remain significant in the multivariate analyzes are relevant to RTW. We primarily wished to examine substantively and statistically what it could mean if some of these variables eventually remained significantly controlled for possible confounders. In a series of multivariate logistic regression analyses, perceived control over important decisions remained a significant predictor of RTW when adjusted for present work ability and symptom pressure. In other words, control over decisions explains a unique portion of the variance of RTW beyond that explained by work ability and symptoms. That is, some patients may RTW based on higher perceived control over important decisions, despite their symptoms and reduced work ability. Similarly, job promotion was an independent predictor of RTW adjusted for symptoms and expected work ability. Consequently, the perception of having a workplace with career opportunities where education and skills are recognized seems to have a positive impact on RTW beyond that of symptoms and work ability. This may indicate that even patients with a lower degree of expected work ability and stronger symptoms will RTW when they have the prospect of promotion. However, we should be cautious about interpreting these results too far. First, all predictors were measured at the beginning of the treatment and aspects of the patients’ situations that we have not examined here may have changed during their treatment. Second, in multivariable analyses that adjust for other moderate and strong predictors, single variables that initially have a moderate effect on the outcome will be vulnerable to decay as significant predictors. In other words, these results can be explained statistically; therefore, we should be cautious in drawing conclusions about their substantive meaning.

We have interpreted how working conditions may affect RTW by making it easier to perform tasks and by creating engagement in and motivation to work. However, we also wish to propose that these work environmental factors, which are often regarded as resources, may have an inherent pressure from the presence pressure demanded by the job. For example, people in professions with a higher degree of autonomy may also have greater responsibility, which can make long-term sick leave difficult. Thus, pressure to RTW can be the result despite disability due to a common mental illness. Similarly, perceived support from colleagues may indicate a supportive exchange. Consequently, colleague expectations may require presence at work and thus act as presence pressure. Therefore, in some cases, a good working environment may cause someone to RTW at an early stage. If there are no specific adjustments at work related to diagnosis and disability, this may lead to a longer period of sick leave at a later time [40]. Various forms of pressure on employees to work despite their health conditions [41] are known to cause sickness presence [41–43]. Therefore, it would be reasonable to question whether the abovementioned working conditions contain elements that generate attendance pressure. In such cases, a good working environment could act as a trap for some workers. However, when workplace adaptations are made, sickness presenteeism may be beneficial to employee health development because it enables employees to master their work tasks [44]. Organizational adjustments entail changes in work tasks or the provision of alternative tasks so that the job can be performed by sick employees without exacerbating their condition, but hopefully improving their health [44,45]. However, this may be somewhat speculative because no measurements of whether the patients themselves perceived that they were returning to work due to presence pressure were used. Therefore, we recommend that RTW studies explicitly investigate whether various types of presence pressures are manifest and whether specific workplace adaptations have been made.

Based on our findings, we acknowledge the importance of including working environment factors in interventions aimed at RTW. That is, ensuring that these patients have a working environment with important job-related resources such as control, support, recognition, and career opportunities. Certainly, such relationships should be addressed in therapy. However, it may be difficult for patients to change these conditions when they are on sick leave. Therefore, we believe that other agencies and personnel, such as corporate health services, human resources personnel, and managers must be taught and given opportunity to follow up these working conditions, so that employees with CMD are able to perform their work in a satisfactory manner.

### Strengths and weaknesses

It is a strength of this study that we gathered data on sick leave from both the start and the end of treatment. However, it would also have been desirable to obtain official register data on sick leave and medical issues as reported by the participants’ GPs. Simultaneously, we caution against interpreting the study results as reflecting the causal relationships between the working environment conditions and RTW, but principally as a statistical relationship. This is an important limitation of all observational studies where various confounding factors that have not been controlled for may show spurious relationships. For example, we measured experienced working environment at the start of treatment, but these self-reporting measures may have been influenced by anxiety and depression. People with depression may experience their working environment as more negative and people with anxiety may perceive their work environment as more uncertain and threatening.

A corresponding weakness is related to the strength of the findings. Although the results were found to be of moderate nature according to Cohen’s *d*, when we controlled for present work ability, future work ability, and CORE total in separate analyzes, these relationships became considerably weaker. Further, in analyzes not shown here, when controlled for these confounders at the same time, only “job promotion” could explain the variance in the dependent variable. This finding may imply that we may have overlooked crucial working environment factors of importance for successful RTW. Future work ability expectancy was particularly important for RTW, which may imply that future studies should concentrate on working conditions positively associated with this variable and that in prospective analyzes demonstrate statistical strength to reduce the predictive ability of future work ability expectancy on RTW. We also consider that it would be beneficial to examine working conditions associated with motivation for work. In this context, it might be conceivable to focus on work conditions that affect employees’ basic psychological needs for autonomy, mastery of work, and safe relatedness to others, as described by the self-determination theory [46]. In addition, it would be desirable to include measures of work engagement and commitment to examine the assumption that these conditions play an important intermediate role in RTW.

Moreover, it was a limitation that the working conditions only were measured at the baseline. Methodologically, it would be desirable to measure these conditions repeatedly to explore the effect of *changes* in working environment experienced on RTW. However, we would not have received complete data because several of the patients were fully or partly on sick leave during the treatment period and were therefore unable to evaluate their working environments. Another point is that although we did not find any selection or drop out bias according to age, sex, diagnoses, and GAF scores, we cannot rule out that other factors such as distrust, fatigue, motivation, or negative feelings towards surveys have biased our data. For example, we found that 69% of the sample had higher education at college or university level, which is a fairly high number. In Norway, approximately 34.5% of the adult population had completed education at college or university level at the time of the study, while about 50% had this education level in Oslo.

We will also encourage care to be taken in generalizing these findings to other contexts. In Norway, where this study was conducted, employees have a particularly good sick leave absence scheme, with fully paid sick leave for an entire year. In countries with more restrictive schemes, we perhaps would not get the same results and perhaps more employees would return to workplaces with a poorer working environment.
